# Education as a predictor of mental illness familiarity and attitudes in a Muslim community

**DOI:** 10.3389/fpubh.2025.1687430

**Published:** 2026-01-28

**Authors:** Zaiboonnisha Mayet, Lebogang Phiri-Sithole

**Affiliations:** 1 Department of Psychology, University of Johannesburg, Johannesburg, South Africa; 2 Department of Psychology, University of Johannesburg, Johannesburg, South Africa

**Keywords:** attitudes, education, familiarity, Islam, mental illness, mental health

## Abstract

**Background:**

Mental illness is a leading cause of disability in South Africa, where stigma, educational disparities, and cultural beliefs are critical barriers to care. Research has largely overlooked the South African Muslim community, particularly regarding how education influences mental illness familiarity and attitudes within a context where spiritual interpretations may uniquely shape stigma. This study investigated the association between level of education and mental illness familiarity and attitudes toward people with mental illness among South African Muslims.

**Methods:**

Using a cross-sectional study design, we recruited 119 South African Muslim adults (81.5% female; mean age = 30.92, SD = 13.01) using purposive snowball sampling, identifying initial participants through Muslim community groups and social media platforms. Eligibility was open to adults (18+) who identified as Muslim, were South African residents, proficient in English, and reported no history of a psychiatric diagnosis. Participants completed an online survey assessing familiarity with mental illness (FMI scale) and attitudes (Beliefs toward Mental Illness scale). We conducted two separate multiple regression analyses to test whether higher education level was associated with greater familiarity and more positive attitudes, controlling for age, gender, employment status, and income.

**Results:**

Participants reported familiarity primarily through indirect exposure (e.g., 85.7% had observed someone in public). Overall attitudes were neutral-to-positive (M = 52.40, SD = 10.06), yet negative stereotypes about incurability and poor social skills persisted. After adjusting for covariates, higher education level remained significantly associated with familiarity (B = 0.83, 95% CI [0.44, 1.22], ^*^p^*^ < 0.001) but not with attitudes.

**Conclusion:**

We found that higher education is associated with greater mental illness familiarity but not with reduced stigmatizing attitudes in this community. The findings suggest that educational attainment alone is insufficient to counteract stigma potentially rooted in cultural and spiritual belief systems. Public health efforts should complement educational outreach with culturally sensitive interventions, developed in partnership with religious communities, to effectively address deeply held stigmatizing beliefs.

## 1 Introduction

Mental illness, characterized by cognitive, behavioral, and emotional disturbances that impair functioning, is a significant global public health challenge, affecting nearly one billion people and contributing substantially to the global disability burden (1). Within the broader construct of mental health literacy, which includes knowledge, attitudes, and help-seeking competencies (2), familiarity with mental illness (direct or indirect contact) is a key experiential component that can challenge stereotypes and reduce stigma (3, 4). Mental disorders are among the leading causes of disability in South Africa. They rank as the fourth largest contributor to the national burden of disease, largely driven by depression and anxiety disorders. This positions mental health as a critical public health priority, following only HIV/AIDS, neonatal disorders, and interpersonal violence (5, 6). This burden was further exacerbated by the COVID-19 pandemic (7), yet the public mental health system remains critically under-resourced. Historical underfunding, with only 5% of the national health budget allocated to mental health in 2017, has resulted in a substantial treatment gap, where only an estimated 8% of affected individuals seek care (8). This gap is sustained by multifaceted individual and systemic barriers, including stigma, low mental health awareness, socioeconomic inequalities, and severe shortages of mental health professionals (9).

Among these barriers, stigma, a multidimensional construct involving stereotypes, prejudice, and discrimination, remains a critical obstacle to care and social inclusion for persons with mental illness (PWMIs) (10). Stigma is closely linked to mental health literacy, defined as the knowledge and beliefs that facilitate the recognition, management, and prevention of mental disorders (4). In the South African context, structural inequalities, particularly in education, directly impact mental health literacy. The education system, still marked by historical disparities, sees only about 28% of adults aged 25–64 having completed secondary education (11). These educational inequities limit exposure to accurate mental health information, as schools in under-resourced communities often lack mental health programs, and public health campaigns have limited reach among low-literacy populations (12, 13). Consequently, individuals with lower educational attainment demonstrate significantly less mental health literacy and more stigmatizing attitudes. For instance, adults without a complete secondary education exhibit 1.8 times greater odds of endorsing stigmatizing views than their tertiary-educated counterparts (2, 14).

Alongside education, familiarity with mental illness, defined as direct or indirect interpersonal contact with PWMIs, is a well-established protective factor against stigma (15). Familiarity, which ranges from incidental encounters to close relationships, fosters empathy and challenges stereotypes, thereby reducing negative attitudes (16). However, access to such meaningful contact and accurate information is often mediated by socioeconomic factors, including educational level.

The interplay between education, familiarity, and attitudes is further complicated by cultural and religious beliefs. This study focuses on the South African Muslim community, where Islam is the second-largest religion after Christianity, with a diverse community of approximately 1.04 million adherents (17). This community is uniquely characterized by its position as a minority within a multi-racial and multi-religious nation, shaped by a complex history of migration, apartheid, and political transition (18). Their context is distinct from that of Muslim-majority nations, where Islamic frameworks may dominate public life, and from diaspora communities in the Global North, whose challenges often center on integration and secularism (19). Instead, South African Muslims navigate a post-apartheid society where their religious identity intersects with pervasive structural inequities in education, healthcare, and the economy (20). Islamic perspectives often view the self as a holistic integration of mind, body, and soul, where health represents a balance between these elements and illness arises from their disruption (21). Within this framework, mental illness is frequently interpreted through a spiritual lens, attributed to supernatural forces, or seen as a test from God (22). While this provides a meaningful explanatory model, it can also foster negative attitudes and stigma when mental distress is attributed solely to spiritual causes, such as divine punishment or spirit possession (23, 24). Stigma in these communities can manifest as shame, secrecy, and the somatization of symptoms, influencing help-seeking behaviors and interactions with both faith-based and biomedical practitioners (24, 25).

Research examining the factors associated with attitudes toward mental illness in specific religious groups is growing. For instance, studies in Arab Muslim communities have found that higher education is linked to less stigmatizing attitudes (26). Similarly in South Africa, educational attainment is associated with mental health literacy in youth populations (27). However, it is unknown whether education is associated with both familiarity with and attitudes toward mental illness within the specific cultural and religious context of South African Muslims, where spiritual beliefs may uniquely shape these perceptions. This represents a critical gap, as public health strategies effective in one context may not translate to another where religious identity profoundly influences health beliefs. Therefore, this cross-sectional study aims to address this gap by examining the association between level of education and both familiarity with mental illness and attitudes toward PWMIs among South African Muslims. Specifically, we hypothesized that higher levels of education will be associated with greater familiarity with mental illness and more positive attitudes toward PWMIs. The findings of this study will enhance the understanding of how socio-educational and religious factors intersect to influence mental health perceptions. This knowledge is crucial for developing tailored mental health interventions and anti-stigma campaigns for religious communities where biomedical and spiritual health paradigms coexist.

## 2 Method

### 2.1 Study design and population

We conducted a cross-sectional, correlational survey of South African Muslim adults (≥ 18 years) between December 2020 and March 2021, during the COVID-19 pandemic.

### 2.2 Sampling and eligibility

Due to the absence of a sampling frame for the specific South African Muslim population, we utilized a purposive snowball sampling strategy. This non-probability method is a recognized approach for recruiting hard-to-reach populations where traditional random sampling is infeasible, as it leverages existing community networks to enhance trust and participation (28). Eligibility criteria were as follows: (1) 18 years of age or older; (2) proficient in English; (3) self-identify as Muslim; (4) a current resident of South Africa; and (5) no prior or current psychiatric diagnosis. English proficiency was essential, as all study materials (consent form, questionnaire, debriefing) were provided in English, which serves as a lingua franca in South Africa (29). Participants with a psychiatric history were excluded to minimize potential confounding, as personal experiences with mental illness could significantly influence reported attitudes and familiarity.

### 2.3 Sample size

We conducted an *a priori* power analysis using G^*^Power software, version 3.1 (30). For a population of ~900,000 (31), the analysis indicated that a minimum sample size of 128 participants was required to detect a medium effect size (*d* = 0.5) with 80% power at an alpha of 0.05. Pandemic-related constraints limited final recruitment. The final sample consisted of 119 participants, which was deemed adequate for detecting medium-sized effects. The survey was distributed online via a Google Form and disseminated through social media platforms, including WhatsApp, Instagram, Facebook, and Twitter.

### 2.4 Measures

#### 2.4.1 Demographic information

Key demographic variables were selected based on their established associations with mental health stigma in the literature (9, 14). Participants provided information on their age, gender, ethnicity, level of education, employment status, and income.

#### 2.4.2 Level of education assessment

Participants' level of education was assessed with four ordinal categories consistent with classifications used in prior South African studies (32, 33). The categories were: (1) primary education (Grades 1-7 or equivalent), (2) secondary education (Grades 8-12 or equivalent), (3) undergraduate education (including diplomas and bachelor's degrees or equivalent), and (4) postgraduate education (honors degree or higher qualifications).

#### 2.4.3 Familiarity with mental illness assessment

The familiarity with mental illness (FMI) scale was developed by Corrigan et al. (34) to elicit individuals' level of familiarity through interpersonal contact with mental illness/PWMI. The scale consists of 7 items representing different levels of contact with PWMIs, with a response format of “yes” (1) or “no” (0). The sum of all seven items forms an index for mental illness familiarity from zero to seven. Higher scores denote more familiarity with mental illness, while lower scores denote less mental illness familiarity. The FMI scale was selected for this study based on its strong psychometric properties demonstrated in the literature. It operationalizes the experiential dimension of mental health literacy, which complements knowledge-based components (2). The scale's validity is well-established, including robust criterion and predictive validity evidenced by consistent associations with lower stigmatizing attitudes and greater social acceptance (4, 35). We found the scale to be reliable in this context, showing an internal consistency of 0.70. This result corroborates reliability coefficients from earlier international (36) and South African studies (37).

#### 2.4.4 Attitudes toward mental illness assessment

Attitudes about mental illness are cognitive evaluations, beliefs or perceptions regarding mental health disorders, fundamentally shaped by stigma, symptom recognition, blame attribution, and help-seeking decisions (15, 38). These attitudes were assessed using the 21-item Beliefs about Mental Illness (BMI) scale, adapted from Hirai and Clum (39). The BMI scale measured four key domains: dangerousness, poor social skills, shame, and incurability of mental illness. All items were phrased as negative stereotypes, and responses were captured on a 5-point Likert scale ranging from 1 (strongly disagree) to 5 (strongly agree). The total score was calculated as the sum of all items, ranging from 21 to 105, with higher scores indicating more negative beliefs. The content and face validity of the BMI scale are supported by its grounding in established stigma constructs documented in the literature (40, 41). In this study, the scale demonstrated high internal consistency (Cronbach's α = 0.84). This finding aligns with the reliability of similar instruments reported in other non-Western contexts, such as Turkey (41), thereby supporting the scale's utility for the present research.

### 2.5 Data analysis

We used SPSS (Version 29.0) for all statistical analyses (42). There were no variables with missing values, which facilitated easier computation (*N* = 119). Descriptive statistics characterized the sample, with categorical variables presented as frequencies and percentages and continuous variables as means ± standard deviations and ranges, as shown in Tables 1, 2. We operationalized variables as follows: age and attitudes (BMI scale score) were treated as continuous variables. Gender and employment status were coded as binary categorical variables (0 = female, 1 = male; 0 = unemployed, 1 = employed). Education level (1 = primary schooling to 4 = postgraduate degree), income bracket (1 = 0 ZAR to 8 = 31,000+ ZAR [approximately 1,685 USD]), and familiarity with mental illness (FMI scale score) were treated as ordinal variables. We did not perform factor analysis to confirm the scale structure, as both FMI and BMI scales are standardized measures with well-validated factor structures (34, 39). However, the use of these scales in our specific cultural context without confirmatory analysis is acknowledged as a limitation. We examined bivariate associations between education, familiarity, attitudes, and demographic variables using Pearson's ^*^r^*^ for continuous and Spearman's ρ for ordinal variables. A multifactorial ANOVA with *post-hoc* tests provided an initial unadjusted assessment of FMI score differences across education levels. Finally, we tested our primary hypotheses by conducting two separate standard multiple regression analyses to examine the role of education level. The first model had familiarity with mental illness as the outcome variable (H_1_), and the second model had attitudes toward mental illness as the outcome (H_2_). Both models included age, gender, employment status, and income as control variables, with education level specified as the primary predictor variable. This simultaneous entry approach allowed for the examination of the unique association between education level and the outcomes after accounting for the other demographic variables in the model. All statistical assumptions for multiple regression were met; normality of residuals was confirmed via the Kolmogorov-Smirnov test (^*^p^*^ > 0.05), homoscedasticity via visual inspection of residual plots, and a lack of multicollinearity was verified (all Variance Inflation Factors < 2).

**Table 1:** Sociodemographic characteristics of South African Muslim participants 2020–2021 (*N* = 119).

**Variable**	**Category**	***n***	**%**
**Age (years)**	Mean (SD)	30.92	(13)
**Gender**			
	Female	97	81.5
	Male	22	18.5
**Ethnicity**			
	Indian	104	87.4
	Mixed Race	11	9.2
	Black	2	1.7
	Asian	1	0.8
	Other	1	0.8
**Education Level**			
	Undergraduate	51	42.9
	Postgraduate	38	31.9
	High School	26	21.8
	Primary School	4	3.4
**Employment Status**			
	Employed	64	53.8
	Unemployed	55	46.2
**Monthly Income Bracket (ZAR [USD])**			
	0	32	26.9
	1,000 – 5,000 (54 – 272)	24	20.2
	6,000 – 10,000 (326 – 544)	12	10.1
	11,000 – 15,000 (598 – 815)	14	11.8
	16,000 – 20,000 (870 – 1,087)	12	10.1
	21,000 – 25,000 (1,141 – 1,359)	2	1.7
	26,000 – 30,000 (1,413 – 1,631)	5	4.2
	31,000+ (~1,685+)	18	15.1

All values are presented as frequency (*n*) and percentage (%) unless otherwise stated. The USD equivalents for the South African Rand (ZAR) income brackets are provided in parentheses for international comparison, based on an approximate exchange rate of 1 USD = 18.37 ZAR (average for March 2022; The World Bank, 2022).

**Table 2:** Frequencies for familiarity with mental illness (*N* = 119).

**Familiarity Item**	***n***	**Yes (%)**
I have observed, in passing, a person I believe may have had a mental illness.	102	85.7
I have a relative who has a mental illness.	75	63.0
A friend of the family has a mental illness.	67	56.3
I have observed PWMIs on a frequent basis.	50	42.0
I have worked with a person who had a mental illness at my place of employment.	48	40.3
My job involves providing services/treatment for PWMIs.	26	21.8
I live with a person who has a mental illness	25	21.0

All values are presented as frequency (n) and percentage (%) unless otherwise stated. PWMIs, Persons with mental illness.

## 3 Results

### 3.1 Participant characteristics

A total of 150 individuals accessed the online survey. After assessment of eligibility, 31 participants were excluded for not meeting the inclusion criteria. The remaining 119 eligible participants all completed the survey, yielding a final analytical sample of 119. The sample recovery rate was 79.3%, representing the proportion of survey initiations that yielded complete, eligible data. Participants had a mean age of 30.92 years (SD = 13), predominantly 81.5% female, and 87.4% Indian. A majority (74.8%) held tertiary education qualifications and 53.8% were employed. About 27% of participants reported no income, nearly half earned less than R10,000 (approx. 544 USD), and a smaller but notable percentage (15.1%) earned R31,000 (approx. 1,685 USD) or more per month (Table 1).

### 3.2 Familiarity with mental illness

As detailed in Table 2, participants' familiarity with mental illness varied across contexts. The majority of participants (85.7%) reported observing someone with a mental illness in public, 63.0% reported knowing a relative with a mental illness, and 56.3% knew a family friend with a mental illness. More direct, involved forms of contact were less frequent: 42.0% had frequently observed someone, and 40.3% had worked with a PWMIs. The least common experiences were those requiring the highest levels of proximity and responsibility, with only 21.8% having provided professional mental health services and 21.0% having lived with someone with a mental illness.

### 3.3 Attitudes toward mental illness

Descriptive statistics for attitudes are presented in Table 3. The mean total score on the BMI scale was 52.40 (SD = 10.06) on a possible range of 21 to 105, suggesting neutral to positive attitudes overall (where higher scores indicate more negative attitudes). Analysis of the subscales revealed a nuanced pattern. On the Dangerousness subscale (range: 6–30), participants' average score was 12.30, indicating general disagreement with the perception that PWMIs are dangerous. Similarly, the average score on the Shame subscale (range: 4–20) was 11.69, reflecting neutral attitudes. In contrast, participants, on average, endorsed more negative views on the Incurability (Mean = 14.33, range: 4–20) and Poor Social Skills (Mean = 14.06, range: 7–35) subscales.

**Table 3:** Descriptive statistics for attitudes toward mental illness and its subscales (*N* = 119).

**Variable**	***M***	**SD**	**Range**
**Mental Illness Attitudes (Total Score)**	52.40	10.06	24 – 78
**Subscale Scores**			
Incurability of mental illness	14.33	3.39	5 – 22
Poor interpersonal skills of PWMIs	14.06	3.19	7 – 23
Dangerousness of PWMIs	12.30	2.89	6 – 20
Mental illness shame/embarrassment	11.69	2.85	6 – 20

M and SD represent mean and standard deviation, respectively. PWMIs = Persons with mental illness. Theoretical score ranges: total scale = 21 to 105; subscales—Dangerousness: 6–30, Poor Social Skills: 7–35, Shame: 4–20, Incurability: 4–20. For all scales, higher scores reflect more negative attitudes.

### 3.4 Factors associated with mental illness familiarity

Bivariate analyses examined the unadjusted associations between key sociodemographic variables (age, gender, education level, income bracket, employment status), familiarity with mental illness, and attitudes toward mental illness. The analyses employed Spearman's rank-order correlations (ρ) for ordinal and non-normally distributed continuous variables, and point-biserial correlations for binary variables (gender, employment status). The complete correlation matrix is presented in Table 4. Familiarity with mental illness was positively correlated with education level (ρ = 0.35, ^*^p^*^ < 0.001). Attitudes toward mental illness were negatively correlated with age (ρ = −0.15, ^*^p^*^ = 0.048). We also observed a significant negative correlation between gender (coded as 0 = female, 1 = male) and income bracket (ρ = −0.19, ^*^p^*^ = 0.012), indicating that male participants reported higher income brackets than female participants.

**Table 4:** Bivariate correlations among study variables (*N* = 119).

**Variable**	**1**	**2**	**3**	**4**	**5**	**6**	**7**
1. Age	—						
2. Gender	−0.06	—					
3. Education Level	0.24^**^	−0.02	—				
4. Income Bracket	0.52^**^	−0.19^*^	0.41^**^	—			
5. Employment Status	−0.32^**^	0.14	−0.31^**^	−0.69^**^	—		
6. Familiarity	−0.03	0.15	0.35^**^	0.05	−0.10	—	
7. Attitudes	−0.15^*^	0.10	0.00	−0.06	0.02	−0.04	—

Gender (0 = Female, 1 = Male) and Employment Status (0 = Unemployed, 1 = Employed) were analyzed as binary variables. Education Level and Income Bracket were treated as ordinal variables. A combination of Pearson and Spearman correlations is reported, as appropriate for variable types. ^*^p^*^ < 0.05, ^**^p^*^ < 0.01.

The independent associations of gender, employment status, education level, and income bracket with familiarity scores were assessed using a multifactorial ANOVA. The association between the continuous variable, age, and familiarity was assessed separately. The results of these analyses, including F-statistics, degrees of freedom, p-values, and effect sizes for all factors in the ANOVA model, are presented in Table 5. The multifactorial ANOVA revealed a statistically significant main effect of education level on familiarity scores, [*F* (3, 115) = 5.65, ^*^p^*^ = 0.001], with a medium-to-large effect size (partial η^2^ = 0.12). This finding was corroborated by Bayesian estimation, which showed a pattern of increasing familiarity scores from primary to postgraduate education levels (see Supplementary Table S1 for complete posterior means and credible intervals). No other main effects for gender, employment status, or income bracket were statistically significant (all ^*^p^*^ > 0.05). Furthermore, the model showed no significant interaction effects.

**Table 5:** Multifactorial analysis of variance and Bayesian estimates for familiarity by demographic factors.

**Variable**	***SS***	***df***	***MS***	***F***	***p*-value**	**Partial η^2^**	***BF***	**Levels**	**Posterior mean [95% CI]**
Gender	8.93	1	8.93	2.52	0.115	0.02	0.25	Male	2.73 [1.93, 3.52]
								Female	3.43 [3.06, 3.81]
Employment	4.58	1	4.58	1.28	0.260	0.01	0.14	Employed	3.48 [3.02, 3.95]
								Unemployed	3.09 [2.59, 3.60]
Education	54.35	3	18.12	5.65	< 0.001	0.12	3.02	Level 1	1.50 [−0.27, 3.27]
								Level 2	2.69 [1.99, 3.40]
								Level 3	3.42 [2.85, 3.99]
								Level 4	4.18 [3.61, 4.76]
Income	18.70	7	2.67	0.73	0.644	0.03	0.00	Bracket 1	3.06 [2.39, 3.73]
								Bracket 2	3.29 [2.52, 4.06]
								Bracket 3	3.33 [2.24, 4.43]
								Bracket 4	3.50 [2.49, 4.51]
								Bracket 5	3.50 [2.41, 4.59]
								Bracket 6	6.00 [3.33, 8.68]
								Bracket 7	2.80 [1.11, 4.49]
								Bracket 8	3.28 [2.39, 4.17]

SS, sum of squares; MS, mean square; BF, Bayes factor; CI, credible interval. Education levels range from 1 (primary schooling) to 4 (postgraduate degree). Income brackets range from 1 = 0 ZAR (0 USD) to 8 = 31,000+ ZAR (~1,685 USD). Bayes factors computed using JZS method with standard reference prior.

### 3.5 Regression analyses of familiarity and attitudes toward mental illness

Table 6 presents two multiple linear regression models assessing the association between education level and both familiarity with and attitudes toward mental illness, controlling for demographic covariates. For familiarity with mental illness (H_1_), the overall model was statistically significant [*F* (5, 113) = 4.49, ^*^p^*^ < 0.001], accounting for approximately 17% of the variance (*R*^2^ = 17). After adjustment for age, gender, employment status, and income bracket, a higher education level showed a significant positive association with greater familiarity (*B* = 0.83, 95% CI [0.39, 1.26], ^*^p^*^ < 0.001). None of the other demographic covariates were statistically significant in this model. In contrast, the model for attitudes toward mental illness (H_2_) was not statistically significant [*F* (5, 113) = 0.79, ^*^p^*^ = 0.561; *R*^2^ = 0.03]. After controlling for the same demographic variables, no significant association was found between education level and attitudes (*B* = −0.30, 95% CI [−2.79, 2.19], ^*^p^*^ = 0.811). Similarly, none of the other covariates were significant predictors.

**Table 6:** Multiple linear regression analyses for familiarity with and attitudes toward mental illness (*N* = 112).

**Variable**	**B**	**SE**	***t*-value**	**95% CI**	***p*-value**
**Familiarity**					
Age	−0.01	0.01	−1.19	[−0.04, 0.01]	0.234
Gender (male)	0.65	0.42	1.52	[−0.19, 1.49]	0.130
Education level	0.83	0.22	3.77	[0.39, 1.26]	**< 0.001**
Employment status	−0.42	0.45	−0.93	[−1.31, 0.47]	0.351
Income	−0.06	0.10	−0.62	[−0.28, 0.14]	0.532
**Attitudes**					
Age	−0.13	0.08	−1.56	[−0.30, 0.03]	0.120
Gender (male)	2.47	2.44	1.01	[−2.36, 7.30]	0.314
Education level	−0.30	1.26	−0.24	[−2.79, 2.19]	0.811
Employment status	−0.63	2.58	−0.24	[−5.71, 4.43]	0.805
Income	0.15	0.61	0.25	[−1.06, 1.37]	0.801

Gender reference category: female. Employment status reference category: unemployed. Model specifications: continuous variables (age, income) were mean-centered. Categorical variables coded as gender (0 = female, 1 = male); education level (1 = primary to 5 = postgraduate); employment status (0 = unemployed, 1 = employed); and income bracket (1 = 0 ZAR to 8 = 31,000+ ZAR). See Supplementary material for USD equivalents.

## 4 Discussion

This study examined the association between level of education, familiarity with mental illness, and attitudes toward PWMIs in a South African Muslim community. The central finding indicates that while higher education is associated with greater familiarity, it is not associated with more positive attitudes. This pattern suggests that in this specific cultural-religious context, the pathways to mental health awareness and stigma reduction are distinct, with deep-seated spiritual and cultural beliefs potentially moderating the influence of education on attitudinal change.

The observed association between higher education level and familiarity with mental illness mirrors findings from broader South African and international contexts (2, 12) and affirms its fundamental role as a social determinant of health literacy (4). Formal education builds mental health literacy by providing structural access to information and fostering the critical skills needed to process it. The persistence of this link after adjusting for income underscores education's unique contribution to awareness, independent of general socioeconomic status. This supports the continued integration of mental health education into formal curricula as a viable strategy for increasing knowledge.

In contrast, the finding that level of education is not associated with more positive attitudes offers a more complex and revealing insight. This suggests that while education may enhance certain components of mental health literacy, such as familiarity, it may not sufficiently address deeply held spiritual beliefs that shape attitudes (14, 43). The findings contrast with meta-analytic evidence on the general stigma-reducing effects of contact and familiarity (44). The divergence can be interpreted through the specific cultural lens of the community. As noted in research on other Muslim populations, spiritual explanatory models where mental illness may be viewed as a supernatural affliction or spiritual test, are prevalent and meaningful (45). It is plausible that in such a context, secular knowledge acquired through education operates in a separate cognitive and experiential domain from faith-informed beliefs about illness etiology and character. Consequently, increased familiarity may not effectively challenge the spiritual narratives that underpin specific stigmatizing attitudes.

This interpretation is reinforced by the nature of the most persistent stereotypes: those concerning incurability and poor social skills. These are not generic prejudices but align closely with conceptions of illness as a permanent spiritual flaw or moral failing (3, 23). Therefore, a key implication is that stigma here is deeply woven into the community's cultural and spiritual beliefs. Effective intervention must move beyond generic awareness-raising to collaboratively address and reframe these specific cultural and spiritual beliefs regarding mental illness. This necessitates a collaborative, community-based approach that works with religious leaders to reframe interpretations of mental illness within a framework of compassion, support, and holistic care, thereby aligning health messaging with community values. Furthermore, the moderate amount of variance explained by our models (*R*^2^ = 0.17 for familiarity) highlights the potential role of factors beyond socio-demographics, such as specific cultural and spiritual beliefs, religious engagement, and illness explanatory models. However, the limited explanatory power for attitudes (*R*^2^ = 0.03), also suggests that stigmatizing attitudes are likely shaped by deeply internalized belief systems and contextual factors not captured by broad socio-demographic categories. Consequently, integrating these cultural and spiritual variables into predictive models offers improved understanding of their unique and interacting contributions.

The predominantly observational and indirect nature of familiarity reported by participants provides another layer for strategic intervention. Theoretical models indicate that indirect contact is less effective than direct, personal interaction in fostering empathy and reducing prejudice (46, 47). Future public health initiatives could therefore be strengthened by designing programs that complement educational components with structured opportunities for positive, meaningful contact with individuals who have lived experiences of mental illness.

### 4.1 Limitations

Several limitations qualify our findings. First, the cross-sectional design prevents causal inference, and the small, non-probability sample restricts broader generalization. Second, we used validated scales (FMI and BMI) but did not confirm their structural validity within this specific cultural-religious sample. Third, self-report measures carry a risk of social desirability bias, and our online recruitment method may have excluded individuals with limited digital access, potentially biasing the sample toward higher socioeconomic status. Fourth, the modest *R*^2^ values show that our socio-demographic model does not fully explain familiarity or attitudes, reflecting the unaccounted influence of cultural/spiritual factors and the complex nature of stigma in this community. Finally, collecting data during the COVID-19 pandemic may have influenced mental health awareness and distress levels, which could have altered familiarity scores and attitudes, thus limiting generalizability beyond the pandemic context.

### 4.2 Future research

These limitations point to clear directions for future research. Subsequent studies should test the psychometric properties of stigma measures within specific religious and cultural groups. Researchers should integrate direct, quantitative measures of cultural and spiritual constructs, such as religiosity, spiritual explanatory models, and community norms, into predictive models to better explain attitudinal outcomes. Longitudinal designs could examine causal pathways between education, familiarity, and stigma over time. Comparative studies across different South African religious and cultural communities would help assess the generalizability of these findings. Finally, future research should develop and evaluate interventions co-designed with religious leaders and community members, integrating mental health literacy with spiritual reframing to effectively reduce stigma.

## 5 Conclusion

In summary, our findings indicate that, within the South African Muslim community, higher education is associated with greater familiarity with mental illness but not with more positive attitudes toward it. This indicates that, in contexts where health beliefs are deeply interwoven with religious worldviews, combating stigma requires a nuanced, two-pronged strategy. Culturally congruent interventions must leverage educational structures to improve mental health literacy while simultaneously partnering with cultural and religious frameworks to address the belief systems that sustain stigma. This two-pronged strategy is essential for achieving genuine mental health inclusion that respects and integrates the community's unique perspective.

## Data Availability

The raw data supporting the conclusions of this article will be made available by the authors, without undue reservation.
