# Supplementary material for: Education as a predictor of mental illness familiarity and attitudes in a Muslim community
Source: Front Public Health. 2026 Jan 28;13:1687430. doi: 10.3389/fpubh.2025.1687430 (PMC12891077; doi:10.3389/fpubh.2025.1687430)
Supplement: Supplementary file 1 [file Table_1.docx]

Supplementary Material

# Supplementary Data

**Supplementary Table S1. Bayesian posterior estimates for education levels**

| **Education level** | **Description** | **Posterior mean** | **95% Credible interval** |
| --- | --- | --- | --- |
| Level 1 | Primary schooling | 1.50 | [-0.27, 3.27] |
| Level 2 | Secondary education | 2.69 | [1.99, 3.40] |
| Level 3 | Undergraduate degree | 3.42 | [2.85, 3.99] |
| Level 4 | Postgraduate degree | 4.18 | [3.61, 4.76] |
